# Peripersonal space representation develops independently from visual experience

**DOI:** 10.1038/s41598-017-17896-9

**Published:** 2017-12-15

**Authors:** Emiliano Ricciardi, Dario Menicagli, Andrea Leo, Marcello Costantini, Pietro Pietrini, Corrado Sinigaglia

**Affiliations:** 1 MOMILab, IMT School for Advanced Studies Lucca, I-55100 Lucca, Italy; 2 Research Center “E. Piaggio”, University of Pisa, Pisa, I-56100 Italy; 3 Department of Neuroscience and Imaging and Clinical Science, University G. d’Annunzio, Chieti, I-66100 Italy; 4 Institute for Advanced Biomedical Technologies - ITAB, Foundation University G. d’Annunzio, Chieti, I-66100 Italy; 5 Centre for Brain Science, Department of Psychology, University of Essex, Colchester, UK; 6 Department of Philosophy, University of Milan, via Festa del Perdono 7, I-20122 Milano, Italy; 7 CSSA, Centre for the Study of Social Action, University of Milan, Milan, I-20122 Italy

## Abstract

Our daily-life actions are typically driven by vision. When acting upon an object, we need to represent its visual features (e.g. shape, orientation, etc.) and to map them into our own peripersonal space. But what happens with people who have never had any visual experience? How can they map object features into their own peripersonal space? Do they do it differently from sighted agents? To tackle these questions, we carried out a series of behavioral experiments in sighted and congenitally blind subjects. We took advantage of a spatial alignment effect paradigm, which typically refers to a decrease of reaction times when subjects perform an action (e.g., a reach-to-grasp pantomime) congruent with that afforded by a presented object. To systematically examine peripersonal space mapping, we presented visual or auditory affording objects both within and outside subjects’ reach. The results showed that sighted and congenitally blind subjects did not differ in mapping objects into their own peripersonal space. Strikingly, this mapping occurred also when objects were presented outside subjects’ reach, but within the peripersonal space of another agent. This suggests that (the lack of) visual experience does not significantly affect the development of both one’s own and others’ peripersonal space representation.

## Introduction

In our daily life, actions are typically driven by vision. When acting upon an object we need to represent its visual features, such as shape, size, and orientation^1–4^, and to map them onto our own peripersonal space, that is, the space immediately surrounding our own bodies and reachable by our limbs^5^. Although this space mapping has been largely investigated over the last two decades^6^, it is still far from clear *whether* and *how* visual experience affects peripersonal space representation. Indeed, what happens to people who have never had any visual experience? How can they map object features into their own peripersonal space? Do they represent this space differently from sighted agents? Or are their peripersonal space representations similar?

Several studies in non-human primates and humans demonstrated that peripersonal space representation could be driven by stimuli other than the visual ones. For instance, ventral premotor^7^ and posterior parietal^8^ neurons, which are typically involved in actions upon reachable targets, may selectively respond to both visual and auditory stimuli when presented within monkey’s reach. Analogously, the presence of an auditory-driven peripersonal space representation has been also demonstrated in sighted humans. A series of TMS studies on motor and premotor cortices showed that auditory stimuli may modulate cortical excitability when presented close to specific agent’s body part only^9–12^.

These findings indicate that auditory inputs can vicariate the visual ones in space mapping. This could explain, partially at least, why congenitally blind people show level of performance in object localization and manipulation comparable to sighted agents^13–16^. However, some studies argued and provided evidence that visual experience may exert a dominant role in the representation of space, even affecting the auditory spatial maps, which might be involved in action planning and control^17,18^. These studies would seem to support the hypothesis that congenitally blind people represent their space differently from sighted agents^18–21^.

To tackle the question as to whether peripersonal space representation can develop independently from (the lack of) visual experience, we carried out a series of behavioral experiments by taking advantage of a spatial alignment paradigm and scrutinizing the space representation of both sighted and congenitally blind people. The spatial alignment effect refers to a decrease of reaction times when subjects perform an action (e.g., a reach-to-grasp pantomime) congruent with that afforded by a presented object (e.g., a mug)^22^. Our group previously demonstrated that this effect could be systematically used to investigate peripersonal space representation, in the visual domain at least. Indeed, the spatial alignment effect has been shown to occur when the affording objects were visually presented within the agent’s reach only^23–25^. Furthermore, this effect turned out to be also modulated by manipulations of the peripersonal space, such as those induced either by the introduction of a barrier preventing subjects from reaching otherwise close objects, or by the use of a tool allowing them to reach and grasp otherwise far objects^25^. These manipulations have been demonstrated to identify the nature and the range of peripersonal space representation^26–29^.

Across Experiments 1, participants had to pantomime a reach-to-grasp action towards a visually cued (in sighted only) or auditorily cued (in sighted and blind subjects) graspable object (e.g. a small ball), presented either within or outside their reach. According to the hypothesis that the peripersonal space representation can develop independently from (the lack of) visual experience, one could expect no differential spatial alignment effect in sighted and blind people. On the contrary, the hypothesis of a visual dominance on space representation would lean towards a difference in the spatial alignment effect between sighted and blind people.

Across Experiments 2, we examined the spatial alignment effect when the same graspable object (a small ball) was auditorily presented close either to the sighted and blind participants or to another individual. In a previous study, we showed that the spatial alignment effect occurred in the visual domain whenever the objects were presented within the peripersonal space of a potential agent, regardless of whether this agent was the participant or another potential actor^30,31^. Is this a result of the visual experience only? Or does something similar occur in the auditory domain too? If the latter were the case, one should expect that both sighted and blind people do not reveal significant differences in mapping not only their own, but also another’s peripersonal space. This would further support the view that the peripersonal space representation can be independent from a specific sensory experience. And this would be not without consequences also for understanding how blind individuals may interact efficiently with other people in a world they have never seen, or so we argued.

## Results

### Experiment 1

When participants had to pantomime a reach-to-grasp movement towards a visually cued or auditorily cued graspable object presented either within or outside agents’ peripersonal space, similar results of a spatial alignment effect were obtained across modalities (i.e., visual and auditory) and groups (i.e., sighted and blind).

**Figure 1:**
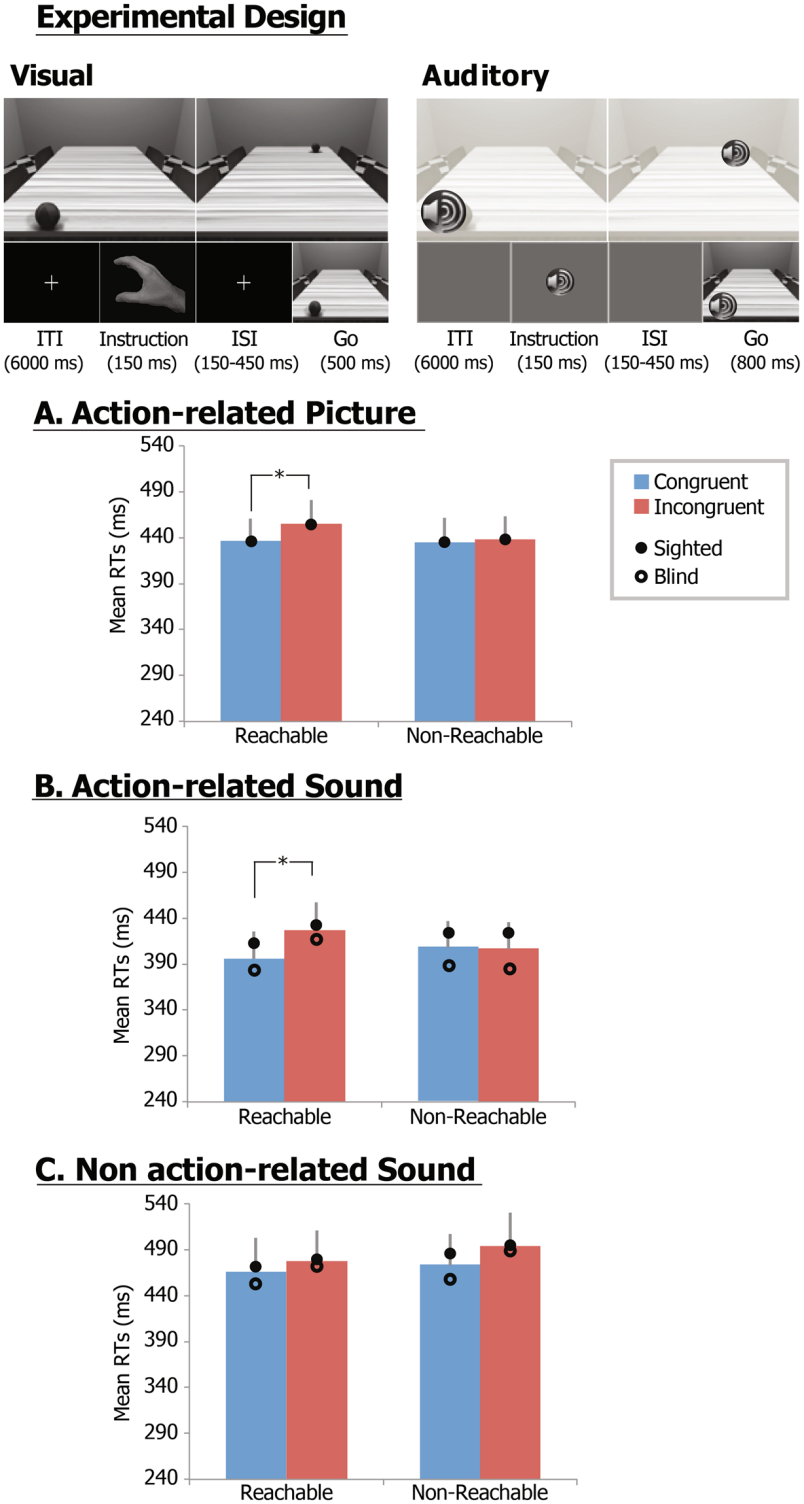
Experimental procedures and results of Experiments 1. Exemplar go stimuli and exemplar trial for the visually-cued Exp. 1A and for the auditorily-cued Exp. 1B and 1C (upper panel). Graphs show estimated marginal mean RTs (±standard errors) for congruent (blue) and incongruent (red) trials of Exp. 1A (**A**), Exp. 1B (**B**) and Exp. 1C (**C**) across sighted and congenitally blind participants; a full circle and an empty circle indicate estimated marginal mean RTs for the sighted and congenitally blind samples, respectively; asterisks highlight significant Location × Congruency interactions and post-hoc T-test comparisons.

First, Exp. 1A aimed at expanding the previously observed spatial alignment effect of our original visual paradigm^23,30^. Participants were requested to replicate a reach-to-grasp pantomime, as prompted by a first instruction stimuli (depicting the hand to be used), once a task irrelevant go-signal (a ball placed on the side of a table which was either congruent or incongruent with the hand to be used) occurred. In half of the trials, the ball was located within the participants’ reach, while in the other half in a non-reachable space (Fig. 1-Experimental design, upper panel). RTs were entered in a linear mixed effects (LME) repeated-measures model with the ‘Location’ of the object (reachable vs. non-reachable) and ‘Alignment’ (congruent vs. incongruent) as within-subjects repeated fixed factors, ‘Subjects’ as random factor and ‘Age’ as covariate. RT analysis revealed a significant Alignment effect (F_1,13_ = 6.9; p = 0.021; partial η^2^ = 0.37) and a Location by Alignment interaction (F_1,13_ = 5.4, p = 0.037; partial η^2^ = 0.29), but no significant Location effect (F_1,13_ = 3.5, p = 0.83). The Alignment effect and interaction were explained by the fact that while RTs to congruent (435 ms) and incongruent (438 ms) trials were comparable in the non-reachable space (p = 0.53), they were significantly higher for incongruent (455 ms) than congruent trials (436 ms, p < 0.001; Bonferroni corrected) in the reachable space (Fig. 1A).

In Exp. 1B, the auditory version of Exp. 1A was employed in both sighted and blind individuals to systematically examine the spatial alignment effect when the reach-to-grasp pantomime was prompted by auditorily instruction stimuli (a beep signal monolateral to the hand to be used) as soon as auditory task-irrelevant go-signal (the sound of a bouncing ball presented on the side either congruent or incongruent with the hand to be used and within or outside the participants’ reach) occurred (Fig. 1-Experimental design, lower panel). The LME - now including the Group (sighted vs. blind) variable as between-subjects fixed factor - showed a significant Alignment effect (F_1,33_ = 13.0; p < 0.001; partial η^2^ = 0.28) and a Location × Alignment interaction (F_1,33_ = 19.8; p < 0.001; partial η^2^ = 0.37). Post-hoc T-test comparisons were performed to assess significant effects and interaction found in the LME analyses. In sighted and blind individuals, the Alignment effect and Location × Alignment interaction were explained by the fact that while RTs to congruent (sighted: 427 ms; blind: 396 ms) and incongruent (sighted: 429 ms; blind: 392 ms) trials were comparable in the non-reachable space (p = 0.79), they were significantly higher (p < 0.001; Bonferroni corrected) for incongruent than congruent trials (sighted: 438 vs. 415 ms; blind: 424 vs. 391 ms) in the reachable space (Fig. 1B). A significant Location × Group interaction was additionally found significant (F_1,33_ = 4.73; p = 0.037; partial η^2^ = 0.13). Neither significant Group (F_1,33_ = 0.2; p = 0.63) and Location (F_1,33_ = 3.2; p = 0.80) effects, nor Alignment × Group (F_1,33_ = 0.1; p = 0.71) nor Location × Alignment × Group (F_1,33_ = 1.4; p = 0.25) interactions were found. While no differences for main effects and interactions between sighted and congenitally blind individuals were found, the Location × Group interaction was explained by the significantly larger RT differences between reachable and non-reachable locations in the blind (difference = 14 ms; p < 0.025; Bonferroni corrected) as compared to the sighted (difference = −1 ms; p = 0.73) group.

Finally, a neutral, action-unrelated environmental sound was presented in Exp. 1C as auditory task-irrelevant go-signal to rule out the possibility that the differences in spatial alignment effect found in the Exp. 1B could be merely accounted by the left-right location of the auditory stimuli, as well as by perceptual/attentional differences in the auditory salience of the presented objects. The LME showed no Group or Location effects, nor significant interactions (Fig. 1C). Only an Alignment effect was found significant (F_1,33_ = 4.4; p = 0.043; partial η^2^ = 0.12), related to the longer RTs for non-reachable (481 ms) as compared to reachable (465 ms) locations across both experimental groups.

### Experiment 2

**Figure 2:**
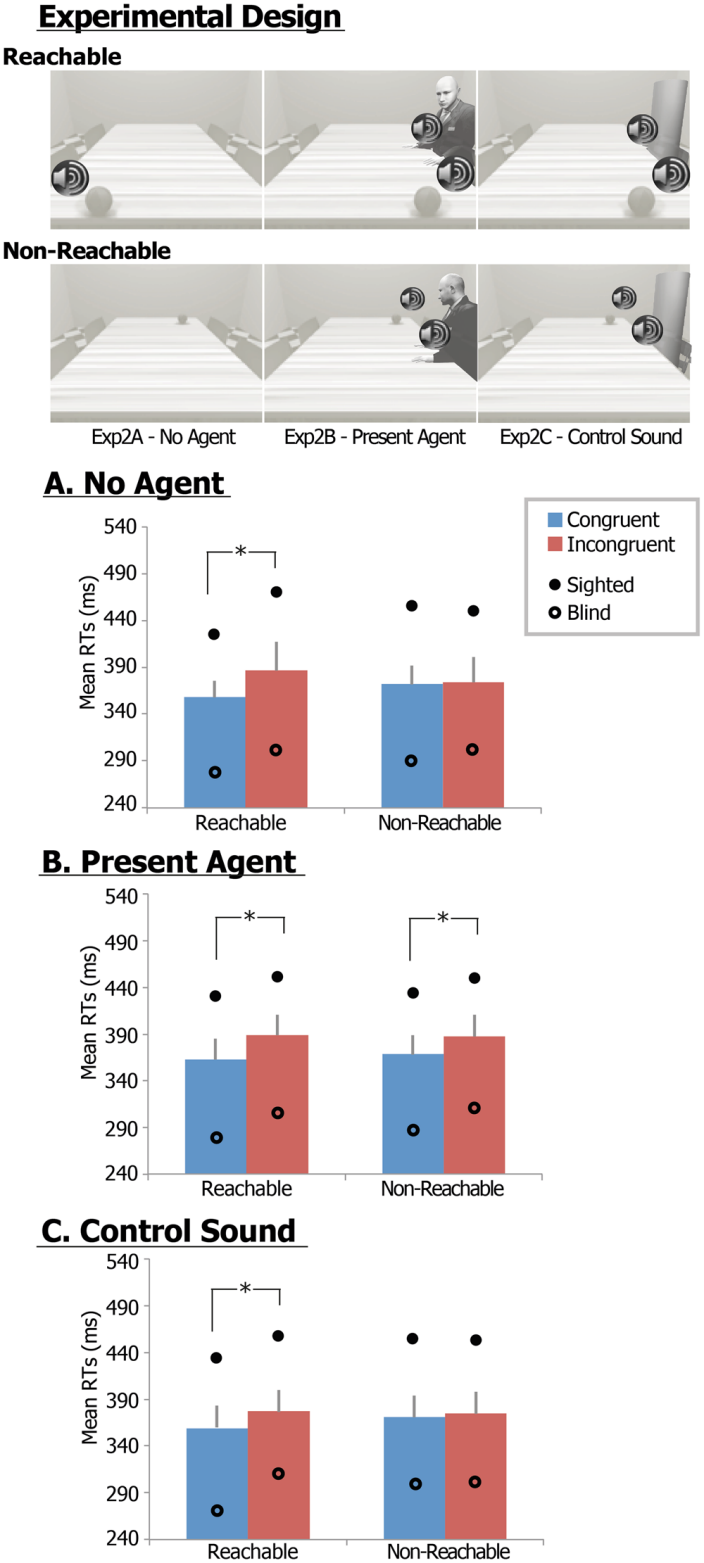
Experimental procedures and results of Experiments 2. Exemplar go stimuli and exemplar trial for Exp. 2 (upper panel); (**A**) Estimated marginal mean RTs of Exp. 2A, (**B**) Exp. 2B and (**C**) Exp. 2C across sighted and congenitally blind participants. In the graphs, error bars indicate standard errors; estimated marginal mean RTs for congruent (blue) and incongruent (red) trials are reported; a full circle and an empty circle indicate estimated marginal mean RTs for the sighted and congenitally blind samples, respectively; asterisks highlight significant Location × Congruency interactions and post-hoc T-test comparisons.

Three sessions within Exp. 2 systematically examined the same spatial alignment effect when an auditorily presented object was located close either to the participants (both sighted and congenitally blind – Exp. 2A) or to another individual (whose presence was simulated by pronouncing the exclamation ‘*hey*’– Exp. 2B) to assess whether the spatial alignment effect occurred whenever the object was presented within the peripersonal space of a potential agent, regardless of whether it was the participant’s own or another’s space (Fig. 2, upper panel), consistently with our previous findings in the visual domain^30,31^. Finally, an artificial neutral sound substituted the agent revealing exclamation in a control condition (Exp. 2C), to exclude that any difference in spatial alignment effects in the Exp. 2B could be accounted by perceptual/attentional differences in the auditory salience of the presented stimuli.

When no other individual was present (Exp. 2A - ‘No agent’ – Fig. 2A), the LME showed overlapping results with Exp. 1B. A significant Alignment effect (F_1,32_ = 10,7; p < 0.003; partial η^2^ = 0.25) and a Location × Alignment interaction (F_1,32_ = 5.5; p < 0.025; partial η^2^ = 0.15) were assessed. As in Exp. 1B, the Alignment effect was restricted to the reachable space (sighted: 431 vs. 472; blind: 278 vs. 304; p < 0.002), and not in the non-reachable spaces, in line with the Alignment × Location interaction. Only a significant Group effect was additionally found (F_1,32_ = 10.3; p = 0.003; partial η^2^ = 0.24), but neither Location (F_1,32_ = 0.9; p = 0.34) effect, Alignment × Group (F_1,32_ = 0.0; p = 1.0), Location × Group (F_1,32_ = 0.01; p = 0.91), nor Location × Alignment × Group (F_1,32_ = 1.4; p = 0.24) interactions were found significant.

When another individual was present on the scene (Exp. 2B - ‘Present agent' – Fig. 2B), the LME showed a significant Alignment effect (F_1,32_ = 36.2; p < 0.0001; partial η^2^ = 0.54) and a Group effect (F_1,32_ = 8.4; p = 0.007; partial η^2^ = 0.21). Specifically, in sighted and blind individuals, post-hoc analyses showed that when another individual was present on the scene, the Alignment effect in terms of congruence gain was observed both within reachable (sighted: 434 vs. 459 ms; blind: 278 vs. 315, p < 0.01, Bonferroni corrected) and non-reachable (sighted: 438 vs. 457 ms; blind: 291 vs. 318, p < 0.01) spaces. No additional Location × Alignment (F_1,32_ = 0.5; p = 0.49), Location × Group (F_1,32_ = 0.3; p = 0.60), Alignment × Group (F_1,32_ = 1.1; p = 0.30), or Location × Alignment × Group (F_1,32_ = 0.1; p = 0.91) interactions were significant.

In the control condition (Exp. 2C - ‘Control sound’ – Fig. 2C), the LME showed overlapping results with the Exp. 2A (‘No agent’), with a significant Alignment effect (F_1,32_ = 13.6; p = 0.001; partial η^2^ = 0.30) and a Location × Alignment interaction (F_1,32_ = 7.8; p = 0.009; partial η^2^ = 0.20). As in Exp. 2A, the Alignment effect was restricted to the reachable space (sighted: 438 vs. 461; blind: 267 vs. 307; p < 0.001, Bonferroni corrected), and not in the non-reachable spaces, in line with the Alignment x Location interaction. A Group effect was additionally found significant (F_1,32_ = 7.8; p = 0.009; partial η^2^ = 0.20). No significant Alignment × Group (F_1,32_ = 0.6; p = 0.44), Location × Group (F_1,32_ = 0.1; p = 0.75), or Location × Alignment × Group (F_1,32_ = 0.8; p = 0.40) interactions were found, thus demonstrating no differences in the interaction effects between sighted and congenitally blind individuals.

When assessing differences between groups, the post-hoc tests confirmed small, though significant, Group effects in both the ‘No agent’ and ‘Control sound’ conditions, as blind individuals showed overall a shorter RT than sighted individuals (sighted vs. blind: 440 vs. 328 ms; 413 vs. 296 ms, respectively), but not in the ‘Present Agent’ (403 vs. 311 ms).

In order to measure the specific effect of the presence of a potential agent on participants’ own peripersonal space, differences in the RTs between congruent and incongruent trials in the non-reachable space for the three different experimental conditions were entered in a LME repeated-measures model with ‘Experimental condition’ (No Agent, Present Agent, Control Sound) and ‘Group’ as within-subjects repeated fixed factors, ‘Subjects’ as random factor and ‘Age’ as covariate. Differences in RTs analysis revealed a significant Experimental condition effect (F_1,32_ = 4.7; p = 0.017; partial η^2^ = 0.13), but no significant Group effect (F_1,31_ = 0.8, p = 0.37) or a Group by Experimental condition (F_1,32_ = 0.5, p = 0.59). In details, differences in RTs were significantly greater for the ‘Present Agent’ as compared to the ‘No Agent’ or ‘Control Sound’ conditions in both groups.

In order to show the consistency of the spatial alignment effects across sighted and blind participants in the two sets of experiments, we reported single subject data in the Supplementary Figures 1 and 2.

## Discussion

The overall aim of the current study was to investigate whether peripersonal space representation can develop independently from (the lack of) visual experience. Accordingly, we carried out two sets of behavioral experiments taking advantage of a spatial alignment paradigm in order to scrutinize how peripersonal space representation functions in both sighted and blind people. There were three main findings.

First, sighted participants displayed a comparable spatial alignment effect in both the visual and the auditory domains (Exp. 1A and 1B). The effect occurred only when the affording object (a small ball) was both visually and auditorily presented within participants’ reach. Second, interestingly a comparable spatial alignment effect occurred in sighted and blind people when listened to the small ball bouncing close to them (Exp. 1B). Third, and even more interestingly, a spatial alignment effect was found also when the affording object was auditorily presented far from sighted and congenitally blind subjects, provided that the object felt within the peripersonal space of another agent, whose presence was auditorily cued (Exp. 2B). Taken together, these three findings suggest that sighted and congenitally blind people did not differ in representing both their own and another’s peripersonal space.

The first finding extends and strengthens previous studies carried out by our group showing at both behavioral and neuronal level that the spatial alignment effect occurs when affording objects are visually presented within the agent’s reach only^23,25^. The current study demonstrates that this holds in the auditory domain too, suggesting that sighted individuals do not differ in representing peripersonal space when capitalizing on either visual or non visual resources.

The second finding of the current study concerns the occurrence of a comparable space alignment effect in sighted and congenitally blind participants, which speaks for a similar peripersonal space representation in both groups. Previous studies showed that auditory stimuli can be mapped onto one’s own space not differently from the visual ones. For instance, it has been reported that both approaching visual and auditory stimuli enhance the corticospinal excitability^10,11,32^. Similar results have also been obtained comparing the tactile and auditory domains^9^. This indicates that peripersonal space might be represented in sensory domains other than vision. The current study moves a step forward by suggesting that (the lack of) visual experience does not significantly affect the development of peripersonal space representation.

This finding seems to be, apparently at least, in contrast with the visual dominance in space representation, as proposed in Röder *et al*.^17^. Specifically, these authors took advantage of an auditory version of the Simon task by asking sighted, late and congenitally blind participants to press a left or right response key depending on the bandwidth of pink noise bursts presented from either a left or right loudspeaker, alternating between performing the task with uncrossed or crossed hands. The results showed that the Simon effect occurred in both conditions for the sighted and late blind participants; while it was reversed when congenitally blind participants crossed their hands. This reversion was even stronger when the task required an explicit matching of the sound location with the position of the responding hand. This would suggest that visual experience affects space representation by inducing the default use of an external coordinate frame for multisensory action control.

A candidate explanation of the difference between our and Röder *et al*.’s findings points first to the difference between tasks. This explanation seems to be supported by a recent studies demonstrating a clear dissociation between the Simon and the spatial alignment effects^33^. And this also may account for why we did not find any spatial alignment effect with an action-unrelated sound (e.g., a neutral environmental sound - Exp. 1C), even when it was presented with a left-right spatial compatibility with participants’ hand.

The difference in task also reflects a difference in space representation. Röder *et al*.^17^ explicitly used the Simon effect, contrasting the uncrossed and crossed hand conditions, with the aim of investigating the frame of reference which is supposed to be used by default in action control and how visual experience might induce a shift from an internal to an external one. Our aim was different: we explored how the peripersonal space is represented, by manipulating the reachability of action-related visually and auditorily cued objects. No doubt that the external frame reference can be useful in mapping space locations for action control purposes. However, there is substantial evidence that when handling with ready-to-hand objects, the space surrounding one’s own body is primarily represented as an action space^34^. In addition, several studies showed that the boundaries of the surrounding space representation varies with the varying of the range of one’s own action^6^. For instance, a seminal single cell study^27^ showed that the extent of hand-related space representation changed when the monkeys used a rake to retrieve otherwise unreachable pieces of food, shrinking back to its normal extension when the monkeys stopped using the rake and just hold it^35^. Similar results have been obtained in healthy^25,36^ and brain-damaged humans^37–41^.

These findings clearly indicate that the peripersonal space primarily functions as a space for action. Our study demonstrates that such action space representation might be developed independently from (the lack of) visual experience. This could explain why congenitally blind people may efficiently handle with a surrounding world they have never seen^18,19,42^. Indeed, comparable performances to sighted individuals have been even described for object identification and manipulation in the surrounding space^43^. In addition, similarly to sighted individuals, tool use has been shown to extend peripersonal space representation in blind people, where this extension turned out to be dynamically and functionally depending on contextual motor demands^44^. Finally, not differently from sighted individuals, a leftward bias in the representation of spatial extents has been found in congenitally blind people when bisecting horizontal rods^45^.

Our and these findings do not imply that visual experience has no effect on space representation, of course. As already mentioned, blind and sighted individuals may represent the external world relying on different reference frames^17^ and several studies indicated that blind individuals are more inclined to primarily rely on body-centered coordinate system, whereas sighted individuals by default shift to an external frame of reference^18–20,46^. Indeed, it has been shown that visual experience facilitates allocentric spatial representation when people are required to memorize a given array of objects^21^. More recently, visual experience has been demonstrated to critically impact even the localization of static and moving sounds. Sighted individuals resulted more accurate than the blind group in doing this, and this difference could be accounted for to a difference in the frame of reference used (i.e., *allocentric* instead *egocentric*)^47^. Finally, there is also evidence that blind and sighted individuals may capitalize on diversified cognitive strategies^19,20,48,49^. However, all these differences are not in contrast with our main finding of a similar action space representation in congenitally blind and sighted people. When required to act upon surrounding objects, congenitally blind and sighted people do not differ in representing their own peripersonal space. Strikingly, that they do not also differ in representing another’s peripersonal space, as our third finding shows.

The third finding was that the spatial alignment effect occurred in both sighted and congenitally blind participants when the bouncing ball was auditory presented far from them but close to another agent whose presence was also auditorily triggered. As for the sighted participants, this finding expands to the auditory domain what has been previously found in the visual domain only^30,31^. Indeed, the representation of peripersonal space is modulated by the visual presence of another potential agent. By using a very similar spatial alignment paradigm^30^, participants were faster in pantomiming a reach-to-grasp movement toward a congruently than toward a no-congruently oriented affording object (e.g., a handled mug), even when the object was visually presented far from them, provided that it turned out to be visually close to a virtual agent such as an avatar. A neuronal counterpart of this behavioral finding has been obtained by a TMS study carried out by our group^31^. We magnetically stimulated the left primary motor cortex and recorded motor-evoked potentials (MEPs) while participants were presented with a handled mug close either to them or to an avatar. Highest MEPs were found both when the mug was near enough to be actually reachable for the participants and also when it was out of reach for them, provided that it was ready to the avatar’s hand. Interestingly, similar results have been also reported in non-human primates. A single cell study showed that premotor neurons typically involved in encoding affording object features (e.g., canonical neurons) usually responded to objects presented within monkey’s reach. A portion of these neurons has been recorded also to respond to objects presented outside the monkey’s reach, provided that these objects were close to another agent’s hand^29^.

Blind participants showed significantly faster reaction times as compared to sighted individuals in the second set of experiments. Faster responses of the blind sample were present, even if not significant, also in Exp. 1B. This observation is consistent with several studies previously assessing a significantly reduced auditory reaction time in congenitally blind participants as compared to sighted participants across different tasks (e.g.^50–52^). Nonetheless, due to the limited size of the blind samples and to the shared participation of six blind subjects across both experimental sessions, any further consideration on superiority of performance or compensatory mechanisms in blind individuals could just be partial.

As for the congenitally blind participants, the finding that they do not differ from sighted individuals in mapping objects ready to both their own and another’s hands strengthens the hypothesis that peripersonal space representation might be developed largely independent from visual experience. This hypothesis seems to be also consistent with several studies demonstrating, at the neural level, that space and action might be represented independently from visual experience. Specifically, distinct functional networks processing task-specific information independently from visual experience have been also described in both sighted and congenitally blind individuals for spatial processing and representation in parieto-occipital areas^18,19,48^. Furthermore a large activation overlap in areas of the dorsal pathway has been found for congenitally blind and sighted participants during performance and working memory maintenance of kinesthetically guided hand movements^53^. Congenitally blind and sighted individuals have also been shown to represent reaching and grasping actions by recruiting a very similar dorso-parietal network^54^. This would suggest a visual experience independent development of action representation and control. Interestingly a parieto-premotor network processing action specific information independently from visual experience has been also described in both sighted and congenitally blind individuals during auditory recognition of motor actions^55,56^. Altogether, these findings support the view that brain morphological and functional architectures are mostly selected to develop and work independently from visual experience and that distinct cortical areas are able to build sensory independent representations of action^14–16^. Furthermore, they indicate that these sensory-independent representations are critical not only for dealing with surrounding things but also for interacting with other people.

The third finding of the current study moves a step further in this direction, suggesting that peripersonal space representation does not concern the surrounding space of our body only, but also might be involved in mapping the space around other bodies, thus contributing to bridge the gap, partially at least, between us and others^57^. To this regard, a seminal single cell study recording from the inferior parietal lobule (area VIP) from the macaque brain demonstrated that there were bimodal neurons which responded, not only to tactile and visual stimuli delivered within the surrounding space of the monkey, but also to visual stimuli presented within the surrounding space of the experimenter facing the monkey. Critically, when visual stimuli were presented at the same distance from the monkey but in the absence of the experimenter, the responses disappeared. According to the authors, these neurons might contribute to an interpersonal bodily space mapping which could be relevant for self and other interactions^58^. The current study supports the hypothesis that this interpersonal mapping should be extended to the action domain, highlighting how people might share their own action space representation to map others’ action space^24,30,31^. And even more interestingly, it demonstrates, for the first time, that the development of interpersonal mapping in the action domain is largely independent from (the lack of) visual experience.

## Methods

### Experiment 1

#### Participants

A group of congenitally blind and three groups of sighted healthy adults took part in Experiments 1: 14 sighted (6 M/8 F, mean age 27 ± 6 years) in Exp. 1A; 25 sighted (10 M/15 F, 34 ± 15) in Exp. 1B; 25 sighted (12 M/13 F, 36 ± 15) in Exp. 1C; 10 blind (5 M/5 F, 43 ± 11; age group comparisons: p = 0.11 for Exp. 1B; p = 0.17 for Exp. 1C) in Exp. 1B and 1C. All subjects were right-handed (with the exception of two sighted for Exp. 1B and one blind left-handed participants), had normal auditory acuity (and normal or corrected-to-normal visual acuity for SC). All subjects were drug-free and received an interview to exclude any medical or neuropsychiatric disorder. All visually-deprived participants were blind from birth and had no recollection of any visual memory (only peripheral causes of congenital blindness were admitted - Table SI). Sighted and blind participants gave their informed consent after the procedures had been explained and retained the right to withdraw from the study at any moment. The study was conducted under a protocol approved by the Ethical Committee of the University of Pisa (1616/2003). All experiments were performed in accordance with relevant international guidelines and regulations.

#### Procedure

In Exp. 1A, two sets of visual stimuli were employed. The first set of stimuli included b/w pictures depicting either a right or a left hand pantomiming of a grasping movement. The second set of stimuli included 3D rooms (similar to those used in^23^), with a table and a ball on it, created with 3D Studiomax v.13. In half of the trials, the ball was placed either on the right or on the left side of the table, and within the participants’ reach or in their non-reachable space (Fig. 1, upper panel). In Exp. 1B, two sets of visual stimuli were employed. The first set of stimuli included a neutral beep (800 Hz, duration 150 ms; 32 bit quantization). The second set of stimuli was the sound of a bouncing solid ball (0–24 KHz, 800 ms; 32 bit, stereo), recorded in an anechoic room at two distances from the microphone to reproduce experimental conditions (reachable and non-reachable). Sound stimuli were normalized using Audacity®2.0.5 (http://audacity.sourceforge.net/). The neutral beep was used as the instruction stimulus and was listened either to the right or to the left ear. The sounds of a bouncing ball were used as the go stimulus. These last stimuli comprised four different sounds of the ball that was placed either on the right or on the left side of the table, and when, in half of the trials the ball was bouncing within the participants’ reachable space, while in the other half in the non-reachable space (Fig. 1, upper panel). Participants evaluated stimuli before starting the main experiments: the ‘reachable’ go stimulus was labeled as near and reachable by all participants with an estimated distance of 21 ± 9 cm, while the ‘not reachable’ go stimulus was labeled as far and not reachable with an estimated distance of 150 ± 30 cm. Two sets of stimuli were used in Exp. 1C. The first set of stimuli included a neutral beep (as in 1B) as instruction stimulus. The second set of stimuli was a neutral, action-unrelated environmental sound (blowing wind: frequency range 0–24 KHz, 1,800 ms; 32 bit, stereo, www.soungle.com). Frequency and amplitude were normalized with the ‘go stimulus’ of Exp. 1B (Audacity®2.0.5), while its volume was modified to simulate the reachable and non-reachable conditions and be used as the go stimulus. The environmental sound was listened either to the right or to the left ear, and, in half of the trials, within the participants’ reachable space, while in the other half in the non-reachable space. Visual stimuli were presented by means of a projecting display (Toshiba TLP-780 projector; Native Resolution: 1024 × 768; Aspect Ratio: 4:3 – XGA; FOV ~60° × 40°). Participants seat about 50 cm far from the display. Auditory stimuli were presented by a headphone system (Philips, SHP1900) and participants were blindfolded.

Each trial consisted on the presentation of the instruction stimulus (i.e., image of the grasping hand for the visual condition, beep sound for the auditory conditions) for 150 ms followed, after a variable delay (ISI ranging from 150 to 450 ms), by the go-stimulus (i.e., visual 3D scenes with a ball placed on a side of a table or a monolateral sound of a bouncing ball). ITI was 6,000 ms. Participants were requested to pantomime a grasp-to-reach movement prompted by the first instruction stimuli (image of grasping hand or monolateral beep sound) as soon as the go-stimulus was viewed or listened to (Fig. 1). Thus, congruent trials refer to the condition in which a participant had to pantomime a reach-to-grasp act with either the right or the left hand ipsilaterally, either to the side of the table where a ball was visually or auditorily placed, while incongruent trials refer to the condition in which the responding hand and the visual or auditory target were in opposite hemispaces. At the beginning of each trial, participants rested their index fingers on two response buttons arranged horizontally on a notepad keyboard. Responses were given by lifting the index finger of the response hand and then making the grasping movement as instructed. This allowed us to measure liftoff time (RT - i.e. the time between onset of the go-stimulus and initial hand movement). Each session consisted of 16 trials that were repeated eight times for Exp. 1A and 1B, and four times for Exp. 1C. Therefore, each participant provided with 32 trials (16 for Exp. 1C) per condition (i.e. reachable/congruent; reachable/incongruent; non-reachable/congruent; non-reachable/incongruent). The presentation of the stimuli and the recording of the participants’ responses (in terms of movement onset) were controlled by Presentation software (Neurobehavioral Systems, Inc., Berkeley, CA). Individuals were administered Exp. 1B and 1C paradigms in a randomly alternated manner, to limit any novelty, learning or fatigue bias due to the order of administration.

#### Data analysis

Trials in which subjects failed to respond (e.g. anticipation of movement) were discarded and error rates were compared across conditions with paired T comparisons between conditions (i.e. congruent vs. incongruent and reachable vs. non-reachable). Error rates did not differ across conditions in Exp. 1 in both sighted and blind participants (p > 0.05, Bonferroni corrected). The mean RT of the correct responses was calculated for each condition within session, thus to obtain 8 individual mean RTs for each condition. The RT values of the corrected responses were corrected for possible outliers through trimming. Specifically, responses either shorter than the 5^th^ percentile or longer than the 95^th^ percentile of all 128 or 64 individual RTs were treated as outliers and not considered (i.e. about 10% of the corrected responses)^59^. Due to unequal sample size and unequal sample variance (Levene test, p < 0.05) between experimental groups, RTs were entered in a linear mixed effect (LME) repeated-measures model with the Location of the object (reachable space vs. non-reachable space) and Alignment (congruent vs. incongruent) as within-subjects repeated fixed factors; Subjects as random factor, and Age as Covariate (fixed factor). For Exp. 1B and 1C, data were entered in a LME model, similar to Exp. 1A, but with an additional Group (sighted vs. blind) variable, as between-subjects fixed factor. Post-hoc T-test comparisons were performed on the estimated marginal means when significant effects or interaction were found in the LME analyses (p < 0.05, Bonferroni corrected). Statistical analyses were performed by using IBM® SPSS® Statistics 21.

### Experiment 2

#### Participants

Twenty-five sighted (12 M/13 F, 34 ± 15 years) and 9 blind (5 M/4 F, 42 ± 12 years; group comparison, p = 0.28 - Table SI) healthy participants took part to Exp. 2. All subjects were right-handed (with the exception one blind). All participants gave their written informed consent.

#### Procedure

In order to reproduce an auditory-based experimental condition similar to our previous experiments^30^, two sets of stimuli were used. The first set of stimuli included a neutral beep (as Exp. 1) used as instruction stimulus. The second set of stimuli included three different sounds (Fig. 2, upper panel): a bouncing solid ball, same as Exp. 1B – ‘No agent’ condition; a bouncing solid ball and a simultaneous human voice sound (simulating the presence of a human agent close to the ball pronouncing the exclamation ‘*hey*’; 0–24 KHz, 800 ms; 32 bit, stereo) – ‘Present agent’; a bouncing solid ball and a simultaneous neutral artificial (i.e., mechanical) sound simulating the presence of a non-human object close to the ball (0–24 KHz, 800 ms; 32 bit, stereo) – ‘Control sound’. Each stimuli were modified in its amplitude (Audacity®2.0.5) to simulate the reachable and non-reachable conditions, and was administered either to the right or to the left ear to simulate congruent or incongruent conditions. Presentation of the ball sound with the human voice or the neutral artificial sound and simple presentation were equally divided across all trials. Each time the bouncing ball sound was presented in association to either the human voice or the neutral artificial sound, the two stimuli were presented at the same distance and to the same side. Experimental procedures were identical to Exp. 1. Each session consisted of 96 trials and was repeated four times.

#### Data analysis

Same as Exp. 1B. Error rates did not differ across conditions (p > 0.05). In addition, to specifically demonstrate the effect of the potential agent on one’s own peripersonal space, across tasks, the differences in the RTs between congruent and incongruent conditions in the non-reachable space for the three different experimental conditions were entered in a LME repeated-measures model with ‘Experimental condition’ (No Agent, Present Agent, Control Sound) and ‘Group’ as within-subjects repeated fixed factors, ‘Subjects’ as random factor and ‘Age’ as covariate.

### Data availability

The data that support the findings of this study are available from the corresponding author upon reasonable request.

## Electronic supplementary material

Supplementary Table and Figures
