# Supplementary material for: Peripersonal space representation develops independently from visual experience
Source: Sci Rep. 2017 Dec 15;7:17673. doi: 10.1038/s41598-017-17896-9 (PMC5732274; doi:10.1038/s41598-017-17896-9)

**Peripersonal space representation develops independently from visual experience**

Ricciardi Emiliano^1^, Dario Menicagli^1^, Andrea Leo^1,2^, Marcello Costantini^3,4^, Pietro Pietrini^1^, Corrado Sinigaglia^5,6^

*^1^* MOMILab, IMT School for Advanced Studies Lucca, Piazza San Francesco 19, I-55100 Lucca, Italy

^2^ Research Center “E. Piaggio”, University of Pisa, Pisa, Italy

^3^ Laboratory of Neuropsychology and Cognitive Neuroscience, Department of Neuroscience and Imaging, University G. d’Annunzio, Chieti, Italy & Institute for Advanced Biomedical Technologies - ITAB, Foundation University G. d’Annunzio, Chieti, Italy.

^4^ Centre for Brain Science, Department of Psychology, University of Essex, Colchester, UK

^5^ Department of Philosophy, University of Milan, via Festa del Perdono 7, I-20122 Milano, Italy

^6^ CSSA, Centre for the Study of Social Action, University of Milan, Italy

**Table SI - Characteristics of the blind volunteers enrolled in the study. Blindness onset for all participants was at birth.**

| Gender | Age | Hand | Cause of blindness | Exps |
| --- | --- | --- | --- | --- |
| F | 54 | R | congenital optic atrophy | 1B, 1C, 2 |
| M | 40 | R | retinopathy of prematurity | 1B, 1C |
| F | 34 | R | microphthalmia | 1B, 1C, 2 |
| M | 44 | R | congenital retinal blindness | 1B, 1C |
| F | 51 | R | retinopathy of prematurity | 1B, 1C |
| M | 45 | R | retinopathy of prematurity | 1B, 1C, 2 |
| F | 34 | L | congenital retinal blindness | 1B, 1C |
| M | 37 | R | retinopathy of prematurity | 1B, 1C, 2 |
| M | 64 | R | congenital retinal blindness | 1B, 1C, 2 |
| F | 22 | R | congenital retinal detachment | 1B, 1C, 2 |
| M | 40 | L | retinopathy of prematurity | 2 |
| F | 44 | R | retinopathy of prematurity | 2 |
| M | 39 | R | microphthalmia | 2 |
| M | 37 | R | retinopathy of prematurity | 2 |

**Figure S1 –** Single subject data have been reported for Experiments 1A-B-C through line graphs to show the consistency of the spatial alignment effects across sighted and blind participants. Line graphs show normalized mean reaction times (i.e., mean RTs for each condition have been normalized to the within-subject, across conditions RT mean) for each of the four conditions (‘reachable’ vs. ‘non-reachable’ and ‘congruent’ vs. ‘incongruent’). Lines have been reported as green if RT increases from the ‘congruent’ to the ‘incongruent’ conditions, consistently with a spatial alignment effect; lines have been reported as green if RT increases from the ‘congruent’ to the ‘incongruent’ conditions, inconsistently with a spatial alignment effect. As evident, individuals data appear prevalently as green for the reachable, but not for the ‘non-reachable’, conditions in Experiment 1A for sighted individuals and in Experiment 1B for both sighted and blind individuals. No prevalence is found for the control condition of Experiment 1C.


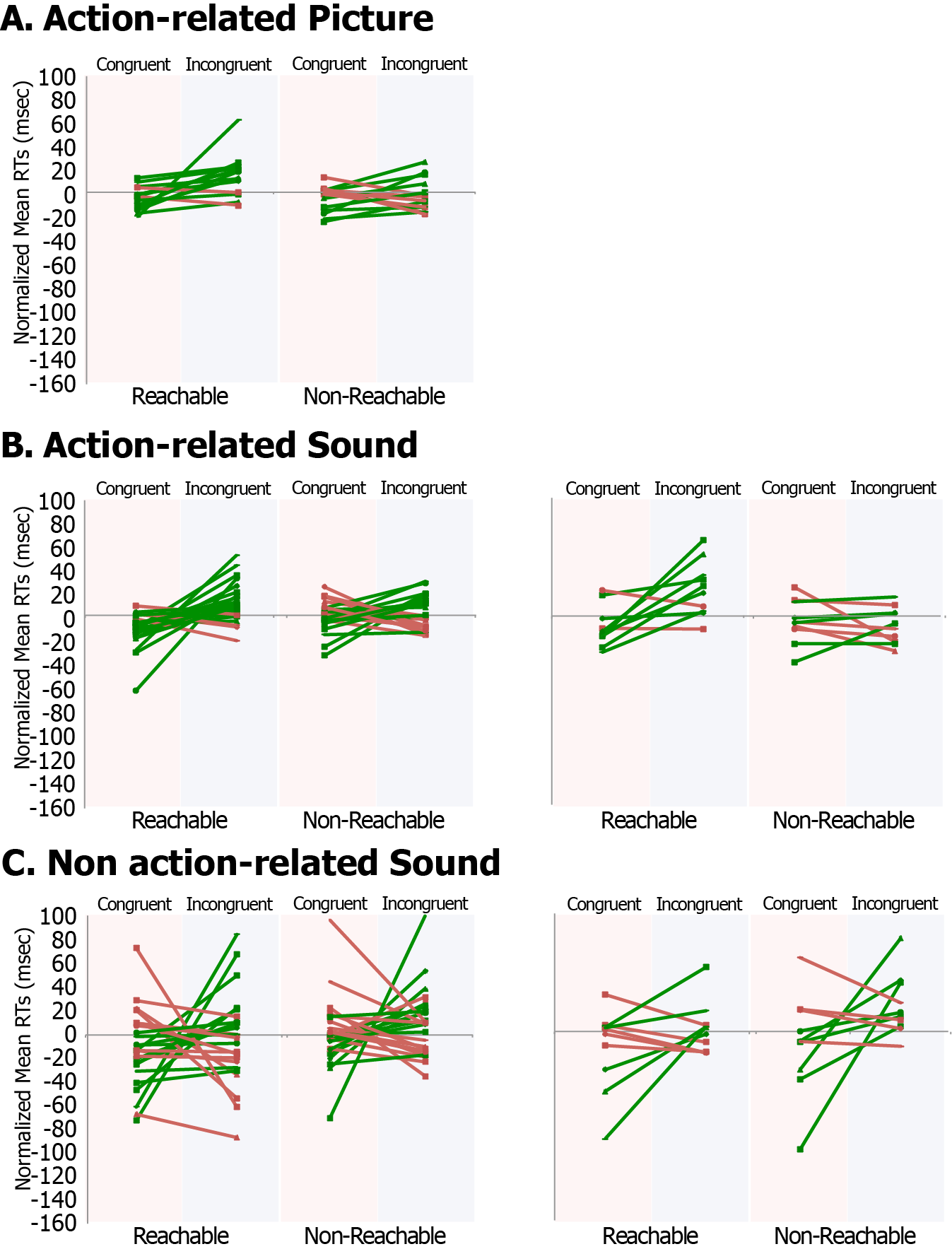


**Figure S2 –** Single subject data have been reported for Experiments 2A-B-C through line graphs to show the consistency of the spatial alignment effects across sighted and blind participants. Line graphs show normalized mean reaction times (i.e., mean RTs for each condition have been normalized to the within-subject, across conditions RT mean) for each of the four conditions (‘reachable’ vs. ‘non-reachable’ and ‘congruent’ vs. ‘incongruent’). Lines have been reported as green if RT increases from the ‘congruent’ to the ‘incongruent’ conditions, consistently with a spatial alignment effect; lines have been reported as green if RT increases from the ‘congruent’ to the ‘incongruent’ conditions, inconsistently with a spatial alignment effect. As evident, individuals data appear prevalently as green for the ‘reachable’, but not for the ‘non-reachable’, conditions in Experiment 2A and 2C, for both sighted and blind individuals, while in Experiment 2B individuals data appear prevalently as green both for the ‘reachable’ and for the ‘non-reachable’ conditions.


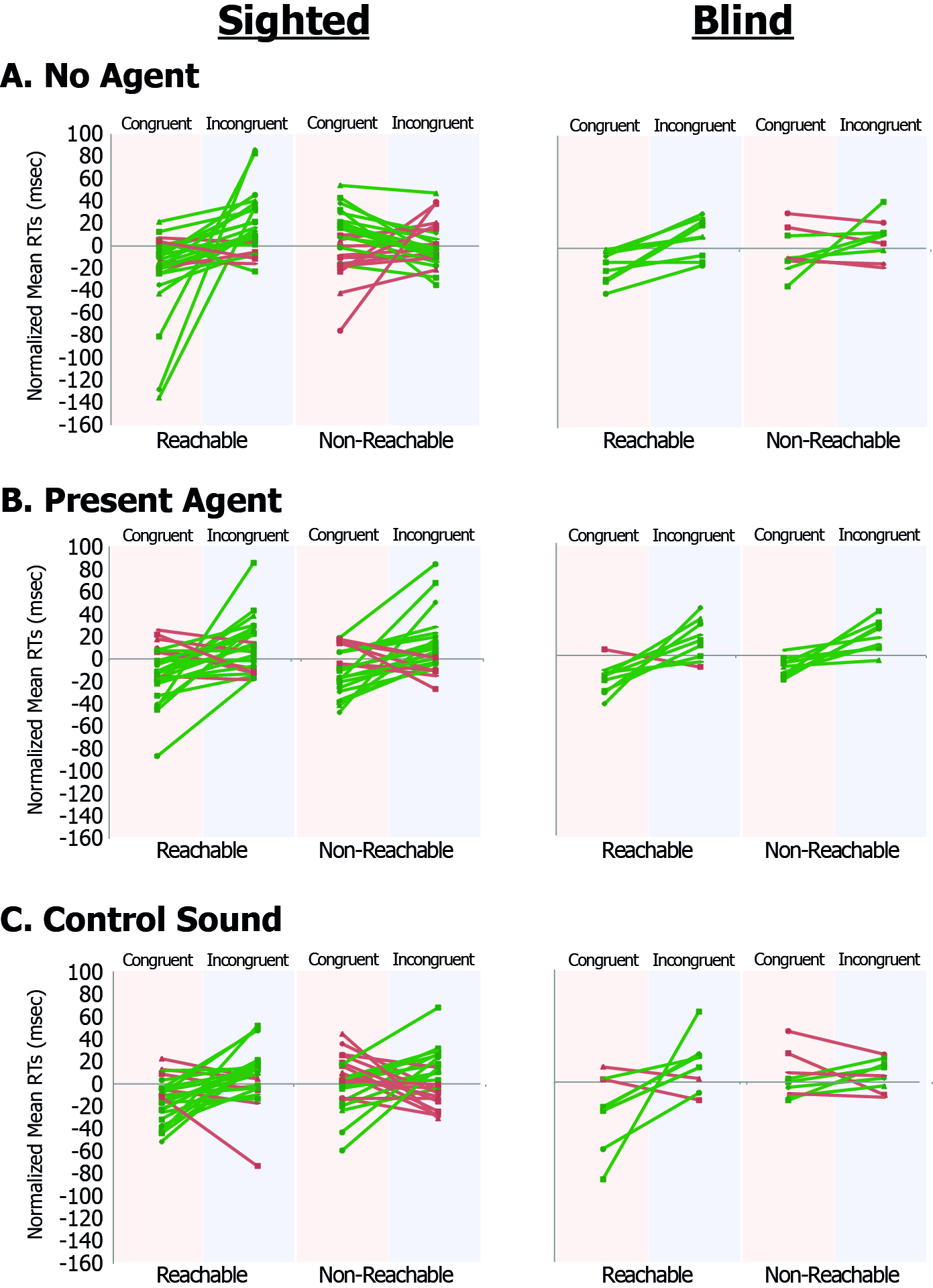

Supplement: Supplementary file 1 — Supplementary Table and Figures [file 41598_2017_17896_MOESM1_ESM.docx]
